# Combined Therapy with Pirfenidone, Metformin, and Mesenchymal Stem Cells Attenuates Bleomycin-Induced Pulmonary Fibrosis in Rats

**DOI:** 10.3390/biomedicines14030642

**Published:** 2026-03-12

**Authors:** Marwa A. Abd Elhamid, Eman T. Mehanna, Noha M. Mesbah, Dina M. Abo-Elmatty, Lubna Jamil, Mohamed M. Hafez

**Affiliations:** 1 Department of Biochemistry, Faculty of Pharmacy, Suez Canal University, Ismailia 41522, Egypt; dr.marwaahmed656@gmail.com (M.A.A.E.); noha_mesbah@pharm.suez.edu.eg (N.M.M.); dina_abouelmouti@pharm.suez.edu.eg (D.M.A.-E.); 2 Department of Histopathology, Faculty of Medicine, October 6 University, Giza 12585, Egypt; lubnajamil.med@o6u.edu.eg; 3 Department of Biochemistry, Faculty of Pharmacy, Ahram Canadian University, Giza 12566, Egypt

**Keywords:** pirfenidone, metformin, mesenchymal stem cells, pulmonary fibrosis, bleomycin, oxidative stress

## Abstract

**Background/Objectives**: Pulmonary fibrosis is a chronic, progressive lung disease marked by scarring and inflammation, leading to impaired respiratory function. This study aimed to investigate the combined therapeutic effects of pirfenidone (PFD), metformin (MET), and bone marrow-derived mesenchymal stem cells (BM-MSCs) on bleomycin (BLM)-induced pulmonary fibrosis in rats. **Methods:** Forty-eight Western Albino rats were divided into six groups: normal control, BLM-positive control, and four treatment groups receiving PFD, MET, BM-MSCs, and their combination. Treatments were administered for four weeks starting on day 21 post-BLM instillation. Lung tissues were analyzed for oxidative stress markers, inflammatory cytokines, apoptotic markers, and fibrogenic gene expression. Histopathological changes were assessed using hematoxylin and eosin (H&E) and Masson’s trichrome staining. **Results:** The combination therapy significantly reduced oxidative stress and inflammatory markers while enhancing antioxidant capacity. It decreased pro-apoptotic Bcl-2-associated X protein (BAX) and increased anti-apoptotic B-cell lymphoma 2 (Bcl-2) levels. Additionally, anti-inflammatory interleukin-10 (IL-10) was elevated, while tumor necrosis factor-alpha (TNF-α) and transforming growth factor-beta 1 (TGF-β1) levels were markedly lowered. Gene expression analysis showed a significant downregulation of matrix metalloproteinase-9 (*MMP-9*) and collagen type 1 alpha 1 (*Col1α1*). Histologically, the combination treatment group exhibited minimal fibrosis and inflammation, closely resembling normal lung tissue. **Conclusions:** The combination of PFD, MET, and BM-MSCs offered superior therapeutic efficacy in treating BLM-induced pulmonary fibrosis compared to individual treatments. This multimodal approach effectively targets oxidative stress, inflammation, apoptosis, and fibrosis, suggesting strong potential for future clinical application.

## 1. Introduction

Pulmonary fibrosis is characterized by significant damage to alveoli, reduced lung capacity, and compromised gas exchange. This condition is believed to result from the infiltration of inflammatory cells and the release of pro-inflammatory substances in the lung tissue. Immune cells such as T cells, B cells, and macrophages play a role in the progression of fibrosis by secreting these substances, which ultimately result in significant lung damage in chronic cases. Important cytokines like transforming growth factor-β (TGF-β), interleukin-1 (IL-1), and interleukin-13 (IL-13) participate in the profibrotic reaction observed in damaged lung areas [1].

Bleomycin exerts its cytotoxic and biological effects through an iron-dependent pathway involving inflammation, apoptosis, oxidative stress and redox-mediated destruction of DNA. The drug forms an active complex of bleomycin Fe^2+^ by complex binding ferrous iron (Fe^2+^). This compound interacts directly with DNA, in the presence of oxygen, inducing DNA damage. Due to such interaction, phosphodiester bonds are broken, and hydrogen is eliminated in the deoxyribose structure, breaking the DNA strands. Also, Fenton-type reactions occur with the bleomycin iron complex to create reactive oxygen species (hydroxyl radicals and superoxide). These ROS promote protein oxidation, fatty acid peroxidation, glutathione loss and additional DNA destruction by increasing oxidative stress [2,3]. Along with genome damage, Bleomycin promotes fibrogenic and inflammatory mechanisms of signaling. This leads to an increase in the expression of cytokines associated with inflammation like TNF-α and IL-1β by stimulating nuclear factor-κB (NF-κB). Simultaneously, Bleomycin increases the cascade phosphorylation of Smad2/3 and the production of transforming growth factor-β1 which increase fibroblast proliferation, epithelial–mesenchymal differentiation, and increased deposition of extracellular matrix [4]. Bleomycin also causes epithelial cells of alveoli to undergo mitochondrial-dependent apoptosis, which can be observed by a higher Bax/Bcl-2 ratio, release of cytochrome c, activated caspase, and perforated membranes of mitochondria. All of these interrelated reactions, the production of oxidative stress, DNA strand rupture, the activation of inflammatory signaling, epithelial death, and fibroblast-induced matrix deposition, are responsible for the toxicity of fibrosis linked to bleomycin administration [5].

Pirfenidone (PFD) is an antifibrotic medication recognized for its anti-inflammatory, antioxidant, and antifibrotic properties. It inhibits TGF-β production and activity, reduces fibroblast proliferation, and modulates collagen synthesis. PFD also alleviates oxidative stress and decreases the expression of IL-1β, a cytokine that promotes the production of fibrogenic substances [6].

Metformin (MET), a robust activator of adenosine monophosphate (AMP)-activated protein kinase (AMPK), has shown promise as an antifibrotic agent. It acts by inhibiting TGF-β production and diminishing Smad2/3 signaling, leading to the downregulation of fibrogenic genes such as collagen 1α1 and 3α1. Additionally, MET aids in reducing oxidative stress by limiting the production of reactive oxygen species (ROS) [7].

Bone marrow-derived mesenchymal stem cells (BM-MSCs) have immunomodulatory and anti-inflammatory characteristics, facilitating lung repair and regeneration. Due to their ability to release anti-inflammatory and antifibrotic cytokines, BM-MSCs are regarded as a promising therapeutic strategy for pulmonary fibrosis [8,9,10].

This study aimed to explore the combined effects of PFD, MET, and BM-MSCs in treating bleomycin (BLM)-induced pulmonary fibrosis in rats, representing the first investigation of this specific combination.

## 2. Materials and Methods

### 2.1. Preparation of BM-MSCs

Halothane inhalation was administered to anesthetize male albino rats that were eight weeks old. After disinfecting the skin using 70% ethyl alcohol, an incision was made. Following dissection, the femur and tibia were immersed in 70% ethyl alcohol for a duration of 1–2 min. The bones were subsequently cleaned in phosphate-buffered saline (PBS) (1X, pH 7.1) within a sterile Petri dish (Hyclone, Logan, UT, USA). Bone marrow extraction was performed in a laminar airflow environment (Unilab biological safety cabinet class II, Jinan, China). Sterilized scissors were used to trim the ends of the long bones, including the femur and tibia. Bone marrow was rinsed out with Dulbecco’s Modified Eagle’s Medium (DMEM) (Lonza, Antwerp, Belgium), which is a culture medium enhanced with 20 mL of complete medium containing 10% (*v*/*v*) fetal bovine serum (FBS) (Lonza, Belgium) and 1% (*w*/*v*) penicillin–streptomycin (Lonza, Bend, OR, USA) [11].

The cultures were kept in an incubator set to 5% humidified CO_2_ at a temperature of 37 °C (Shellac, New York, NY, USA) for a period of 7–10 days. When the cultures achieved 80–90% confluence, they were rinsed twice with PBS and dissociated with 0.25% trypsin-EDTA (Lonza, Belgium) for roughly 5 min at 37 °C. The cells were then centrifuged at 2400 rpm for 20 min, resuspended in complete medium, and placed in Falcon flasks for culture. A single-cell suspension of 1 × 10^6^ cells/mL was created using third-generation cells, which were treated with 2.5 g/L trypsin [11].

Bone marrow-derived MSCs (BM-MSC) were defined by flow cytometry after fluorescent isothiocyanate (FITC) or phycoerythrin (PE) labeling with the corresponding antibodies. Cells were initially gated on side and forward scattered to eliminate debris, and isotype-matched controls were applied to confirm the specificity of antibody binding. BM-MSCs were stained with the respective antibodies for 15 min at room temperature and washed twice with phosphate-buffered saline (PBS). Levels of fluorescence were subsequently quantified by flow cytometry. BM-MSCs were classified as negative for CD45 (BioLegand, San Diego, CA, USA, Cat. No. 202201) and HLA-DR (BioLegand, USA, Cat. No. 327005) and positive for CD105 (BioLegand, USA, Cat. No. 562408), CD90 (BioLegand, USA, Cat. No. 206101), and CD73 (BioLegand, USA, Cat. No. 551123). For isotype controls, staining specificity was checked by using matched FITC- and PE-conjugated isotype antibodies: FITC Mouse IgG2a, _K_ Isotype control (BioLegand, USA, Cat. No. 400103) and Mouse IgG2a, _K_ Isotype control PE (BioLegand, USA, Cat. No. 400107). The analysis was performed using a BD FACSCalibur™ (BD Biosciences, San Jose, CA, USA) that has a 488 nm excitation. Finally, the proportion of positive cells was reported [11,12,13].

### 2.2. Study Design

Forty-eight male Western Albino rats, each weighing around 200 g, were sourced from the National Research Center in Cairo, Egypt. The rats underwent an acclimatization period of approximately seven days, during which they were given food and tap water. The Ethics Committees of the Faculty of Pharmacy at Ahram Canadian University (code # REC1624) and the Faculty of Pharmacy at Suez Canal University (code: 202201MA3) approved the study protocol. The rats were randomly divided, using computer-generated random allocation. Also, body weight was matched to avoid baseline bias, forming the following experimental groups:

Group I (Healthy control group, *n* = 8): These rats received 0.4 mL of saline intratracheally, followed by a daily oral dose of saline for four weeks.

Group II (Pulmonary fibrosis group induced by BLM, *n* = 8): Pulmonary fibrosis was induced by delivering a single intratracheal injection of BLM (brand name: Bleomycin^®^ 30 units/vial, Fresenius Kabi, Lake Zurich, IL, USA) at a dosage of 5 mg/kg body weight in 0.4 mL saline after being anesthetized with ketamine [14].

Group III (BLM-induced pulmonary fibrosis group treated with PFD, *n* = 8): On day 21 following the BLM injection, the rats received PFD (brand name: PIRFENEX^®^ Tablet 200 mg, Optimus Pharma Pvt. Ltd., Telangana, India) orally at a dose of 200 mg/kg per dose, administered twice daily for four weeks. The PFD was suspended in 40 mg/mL carboxymethylcellulose (CMC) [15].

Group IV (BLM-induced pulmonary fibrosis group treated with MET, *n* = 8): On day 21 post-BLM injection, the rats were given MET (brand name: Glucophage^®^ Tablet 500 mg, Merck, Cairo, Egypt) orally at a dose of 100 mg/kg/day for four weeks [16].

Group V (BLM-induced pulmonary fibrosis group treated with BM-MSCs, *n* = 8): On the 21st day after inducing fibrosis, the rats were intravenously administered a single dose of BM-MSCs (2 × 10^6^ cells/rat) in complete medium [8,11].

**Scheme 1:**
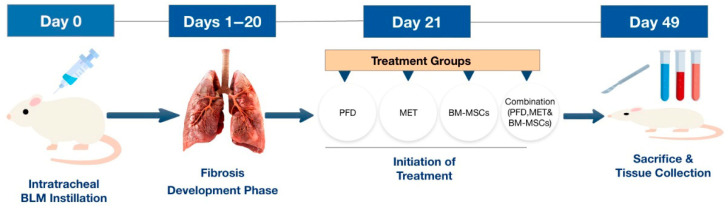
Schematic presentation of animal workflow study.

Group VI (BLM-induced pulmonary fibrosis group treated with PFD, MET, and BM-MSCs, *n* = 8): On day 21 following the BLM injection, the rats received a single dose of BM-MSCs (2 × 10^6^ cells/rat) in complete medium together with a combined treatment of PFD (200 mg/kg per dose, twice daily) and MET (100 mg/kg/day) for four weeks (Scheme 1).

### 2.3. Collection of Samples

At the end of the experiment, rats were euthanized via decapitation while under ketamine anesthesia. The lungs were removed and cut in half with a scalpel. One half of each lung was quickly placed in 10% neutral buffered formaldehyde (Sigma-Aldrich, St. Louis, MO, USA) to maintain tissue structure for subsequent histopathological examination. The samples were then subjected to an automated tissue processor (Leica TP1020, Leica Biosystems, Nussloch, Germany) followed by embedding in paraffin wax with a melting point of 56–58 °C (Sigma-Aldrich, USA). Paraffin blocks were cut with a thickness of 2 μm by means of rotary microtome (Leica RM2235, Leica Biosystems, Germany). Lung tissue samples were stained with the staining reagents of hematoxylin and eosin (H&E) (BioDiagnostic, Giza, Egypt) in addition to Masson’s trichrome staining kit (BioDiagnostic, Egypt) following the standard procedures of the manufacturer. Then, DPX mounting medium (Sigma-Aldrich, USA) was used to stain the slides followed by visualization under light microscope (OlympusBX43, Olympus Corporation, Tokyo, Japan) at the magnification of ×400, and representative photomicrographs were observed with the use of a digital camera system (Olympus DP27, Olympus Corporation, Japan).

The remaining half of each lung was promptly frozen and kept at −80 °C for biochemical evaluation. This analysis involved measuring levels of malondialdehyde (MDA), reduced glutathione (GSH), superoxide dismutase (SOD), nitric oxide (NO), B-cell lymphoma 2 (Bcl-2), Bcl-2-associated X protein (BAX), interleukin-10 (IL-10), transforming growth factor-beta (TGF-β), and tumor necrosis factor-alpha (TNF-α). The expression of mRNA for collagen type I alpha 1 chain (Col1α1) and matrix metalloproteinase 9 (MMP-9) was also investigated in the frozen lung tissues.

### 2.4. Biochemical Measurements

Colorimetric analysis was utilized to quantify the levels of MDA, GSH, SOD, and NO in lung tissue homogenates using Biodiagnostic colorimetric assay kits (Dokki, Giza, Egypt, Cat. No. MD 2528 for MDA, GR 2510 for GSH, NO 2532 for NO, and SD 2520 for SOD). The measurement of TNF-α was performed using a specific rat TNF-α ELISA kit (Cusabio, Houston, TX, USA, Cat. No. CSB-E11987r). The levels of IL-10 and TGF-β were evaluated with the rat IL-10 ELISA kit and the rat TGF-β ELISA kit (MyBioSource, San Diego, CA, USA, Cat. No. MBS764911 and MBS260302, respectively). Markers related to apoptosis, BAX and Bcl-2, were measured using the Rat Apoptosis Regulator (BAX) ELISA Kit and the Rat B-cell CLL/Lymphoma 2 (Bcl-2) ELISA Kit (Cusabio, USA, Cat No. CSB-EL002573RA and CSB-E08854r, respectively).

Using a Teflon homogenizer, the samples of lung tissue were mixed in ice-cold phosphate-buffered saline in a pH 7.4 condition to guarantee effective tissue breakdown at regulated temperatures. Centrifugation was performed on the prepared homogenates for 15 min at 4 °C and 4000 rpm in order to separate and remove cellular debris. Furthermore, the transparent supernatants were properly harvested and utilized for further biochemical studies. Following the manufacturer’s instructions, the Bradford protein assay (BioDiagnostic Kit, Giza, Egypt) was used to measure the amount of protein in each prepared homogenate. Spectrophotometric measurements of absorbance were carried out at 595 nm, and the concentrations of the proteins were determined by a standard calibration curve created using bovine serum albumin (BSA). All measured markers of oxidative stress, inflammatory mediators, and apoptotic markers were standardized to the total content of protein to promote sample comparability, and, when applicable, represented as mmol/g protein.

### 2.5. Quantitative Determination of mRNA Expression of MMP-9 and Col1α1 by Real-Time PCR

Total RNA was isolated from lung tissue utilizing a total RNA extraction kit (ELK Biotechnology, Sugar Land, TX, USA, Cat. No. EP014). The extracted RNA was converted into complementary DNA (cDNA) with the TruScript First Strand cDNA Synthesis Kit (Norgen Biotech Corp, Thorold, ON, Canada, Cat. No. 54420). The expression levels of the genes *MMP-9* and *Col1α1* were normalized against *β-actin*, serving as an internal reference. Each polymerase chain reaction (PCR) mixture, totaling 20 μL, included 3 μL of cDNA template, 1 μL of each primer, 10 μL of PCR Master Mix (Promega, Madison, WI, USA), and 5 μL of nuclease-free water. The PCR cycling parameters were set as follows: an initial denaturation step at 95 °C for 10 min, succeeded by 40 cycles consisting of denaturation at 95 °C for 15 s, annealing for 30 s, and a final extension at 72 °C for 30 s. Real-time PCR was conducted using a StepOnePlus Real-Time PCR Thermal Cycler (Applied Biosystems, Waltham, MA, USA) (Table 1).

The relative quantification method has been described in a straightforward and concise manner using the real-time PCR methodology. Whereas values of Ct were initially determined for target genes (MMP-9 and Col1α1) as well as for internal standard gene (β-actin). To normalize expression of the target gene, the Ct of β-actin was subtracted from the Ct of every single target gene to determine the ΔCt value (ΔCt = Ct_target_ − Ct_β-actin_). The ΔCt of the groups receiving treatment was then compared to that of the standard control group to calculate the ΔΔCt values (ΔΔCt = ΔCt_treated_ − ΔCt_control_). The 2^−ΔΔCt^ technique was used to calculate fold change. In comparison to the normal control group, all gene expression findings are now clearly shown as relative fold change.

### 2.6. Statistical Analysis

The analysis of data was executed using SPSS software (version 20; SPSS, Chicago, IL, USA). Results were presented as mean ± standard deviation (SD). Comparisons between groups were carried out using analysis of variance (ANOVA), accompanied by Bonferroni’s post hoc test for multiple comparisons. The normality of data distribution was first tested through Shapiro–Wilk test and the homogeneity of variances between groups was tested through Levene’s test. The final data analysis was performed after identifying and assessing the outliers via Grubbs’ test. A *p*-value of less than 0.05 was considered statistically significant. The sample size (*n* = 8/group) was decided according to the prior studies based on similar experimental models of pulmonary fibrosis, which provides sufficient statistical power.

## 3. Results

### 3.1. Characterization of BM-MSCs

**Figure 1:**
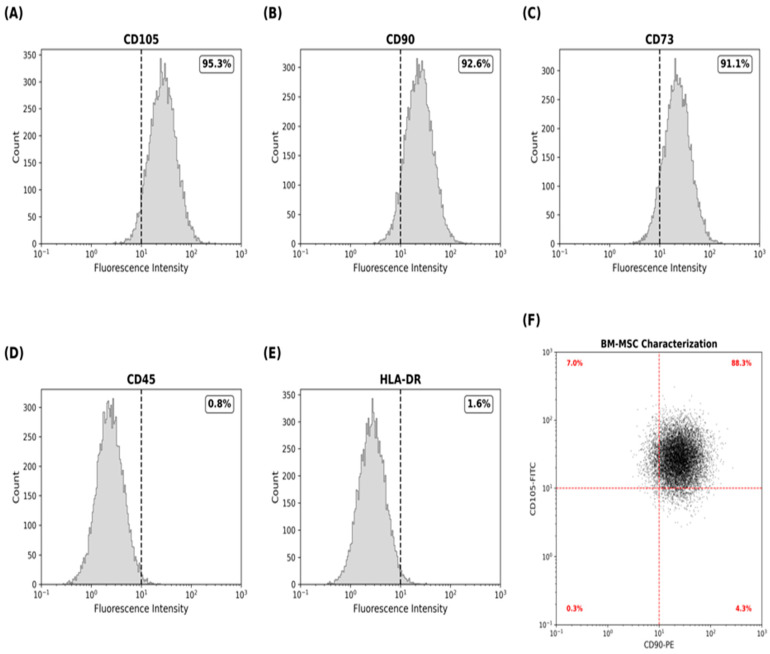
Flow cytometry results regarding characterization of BM-MSCs. Representative histograms show the expression of (**A**) CD105, (**B**) CD90, (**C**) CD73, (**D**) CD45, and (**E**) HLA-DR. The vertical dashed line in each histogram indicates the gating threshold used to distinguish positive from negative cell populations based on the corresponding isotype control. The percentage of positive cells for each marker is indicated in the upper right corner of each histogram. (**F**) A representative dual-parameter dot plot illustrates the co-expression of CD90 (*x*-axis) and CD105 (*y*-axis), with quadrants defined by the gating thresholds. Quadrant analysis shows that 88.3% of the cells are double-positive (CD90^+^/CD105^+^), while 7.0% are CD105^+^/CD90^−^, 4.3% are CD90^+^/CD105^−^, and 0.3% are double-negative.

Flow cytometry confirmed that cultured BM-MSCs maintained their stromal properties. CD105 was observed in 95% of cells, while CD90 and CD73 expressions were more than 90%. There was minimal hematopoietic contamination of the cultures, whereas CD45 expression was below 1% and HLA-DR expression was below 2%, signifying the low immunogenicity of the administered cells (Figure 1).

### 3.2. The Levels of Oxidative Stress Markers in the Lung Tissue of the Experimental Rats

**Figure 2:**
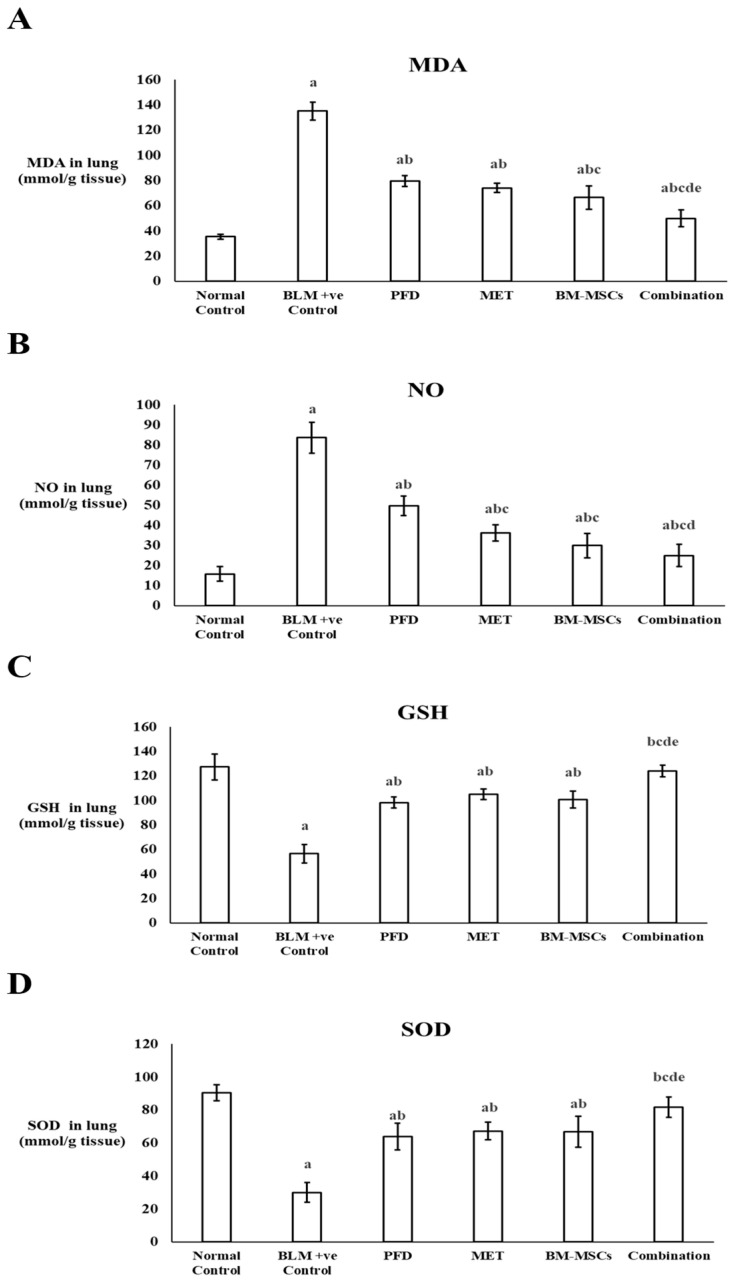
Levels of oxidative stress markers in the lung tissue of the experimental rats. (**A**) MDA, (**B**) NO, (**C**) GSH, and (**D**) SOD. MDA = malondialdehyde; NO = nitric oxide; GSH = reduced glutathione; SOD = superoxide dismutase; PFD = pirfenidone; MET = metformin; BM-MSCs = bone marrow-derived mesenchymal stem cells. The parameters were detected by a colorimetric method. Data are expressed as mean ± SD. Data were analyzed by one-way ANOVA followed by Bonferroni’s post hoc test. Differences were considered significant at *p* < 0.05 (*n* = 8). ^a^ significantly different vs. normal control, ^b^ significantly different vs. BLM-positive control, ^c^ significantly different vs. PFD alone, ^d^ significantly different vs. MET alone, ^e^ significantly different vs. BM-MSC group.

Malondialdehyde (MDA), a recognized indicator of lipid peroxidation, was quantified in the lung tissue of experimental rats. In the BLM + ve control group, MDA concentrations demonstrated a significant increase (*p* < 0.05), exhibiting a rise of 3.8 times compared to the normal control group. Administration of PFD, MET, or BM-MSCs individually led to a reduction in MDA levels by 41.17%, 45.17%, and 50.74%, respectively. The combination therapy resulted in a substantial decrease of 63.08% in MDA levels in lung tissue when compared to the positive control group (*p* < 0.05) (Figure 2A) (Table 2).

Additionally, nitric oxide (NO) concentrations were measured in lung tissues. Administration of BLM led to a 5.2-fold increase in NO levels in comparison to the normal control group. Meanwhile, treatment with PFD, MET, or BM-MSCs alone significantly lowered NO levels by 40.64%, 56.70%, and 64.12%, respectively, compared to the positive control group. The combined treatment with PFD, MET, and BM-MSCs together resulted in a significant reduction in NO levels by 70.22% when compared to the positive control group, which was more effective than the individual administration of each treatment (Figure 2B) (Table 2).

The levels of glutathione (GSH) and superoxide dismutase (SOD), both effective antioxidants, were also assessed in lung tissues. The BLM + ve control group showed a significant decrease in GSH and SOD levels by 55.56% and 66.85%, respectively, compared to the normal control group. Treatment with PFD, MET, or BM-MSCs alone resulted in a significant increase in GSH levels by 1.7-fold, 1.8-fold, and 1.7-fold, respectively. For the SOD biomarker, levels increased by 2.13-fold, 2.24-fold, and 2.23-fold, respectively, after the administration of PFD, MET, or BM-MSCs alone compared to the positive control group (*p* < 0.05). The combined treatment considerably enhanced GSH and SOD levels, demonstrating a rise of 2.18-fold and 2.7-fold, respectively, compared to their levels in the BLM-positive control group (Figure 2C,D) (Table 2).

### 3.3. The Levels of Apoptotic Markers in the Lung Tissue of the Experimental Rats

**Figure 3:**
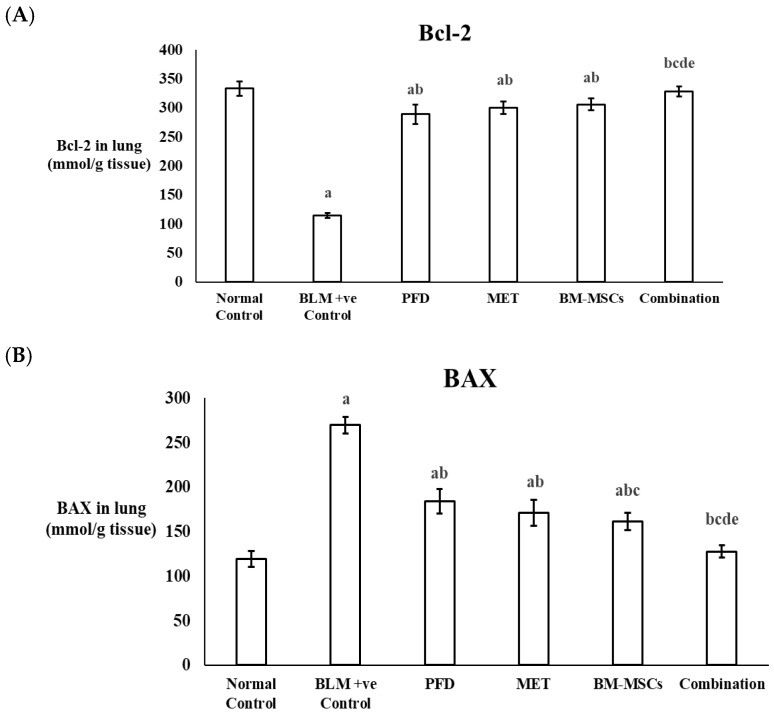
The levels of apoptotic markers in the lung tissue of the experimental rats. (**A**) Bcl-2 and (**B**) BAX. Bcl-2 = B-cell lymphoma-2; BAX = Bcl-2-associated X protein; PFD = pirfenidone; MET = metformin; BM-MSCs = bone marrow-derived mesenchymal stem cells. The parameters were detected by a colorimetric method. Data are expressed as mean ± SD. Data were analyzed by one-way ANOVA followed by Bonferroni’s post hoc test. Differences were considered significant at *p* < 0.05 (*n* = 8). ^a^ significantly different vs. normal control, ^b^ significantly different vs. BLM-positive control, ^c^ significantly different vs. PFD alone, ^d^ significantly different vs. MET alone, ^e^ significantly different vs. BM-MSC group.

The levels of the biomarkers Bcl-2 and BAX were assessed in lung tissues. Following BLM administration, Bcl-2 levels showed a significant decrease of 65.61% when compared to the normal control group (*p* < 0.05). In contrast, Bcl-2 levels were found to be significantly increased by 2.86-fold after the combined treatment with PFD, MET, and BM-MSCs compared to the BLM-positive control group, surpassing the effects of each individual treatment. Notably, the combined therapy restored Bcl-2 levels to match those of the normal control group, with no significant difference detected (Figure 3A) (Table 2).

Regarding the pro-apoptotic marker BAX, levels were elevated by 2.26-fold following BLM administration in comparison to the normal control. Treatment with PFD, MET, or BM-MSCs alone resulted in reductions in BAX levels by 31.87%, 36.59%, and 40.29%, respectively, relative to the BLM-positive control group. Importantly, the combined treatment led to an even greater decrease in BAX levels, achieving a 52.76% reduction compared to the positive control group (Figure 3B) (Table 2).

### 3.4. Levels of Inflammatory Markers in the Lung Tissue of the Experimental Rats

**Figure 4:**
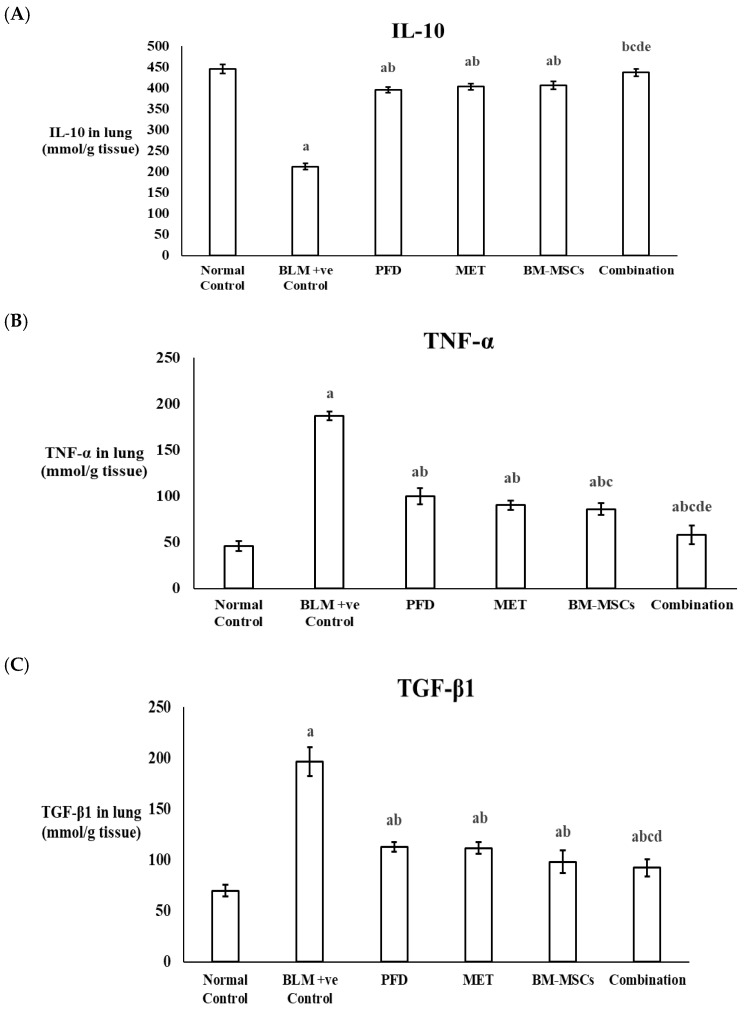
Levels of inflammatory markers in the lung tissue of the experimental rats. (**A**) IL-10, (**B**) TNF-α and (**C**) TGF-β1. IL-10 = Interleukin-10; TNF-α = tumor necrosis factor-α; TGF-β1 = transforming growth factor-beta 1. PFD = pirfenidone; MET = metformin; BM-MSCs = bone marrow-derived mesenchymal stem cells. The parameters were detected by ELISA method. Data are expressed as mean ± SD. Data were analyzed by one-way ANOVA followed by Bonferroni’s post hoc test. Differences were considered significant at *p* < 0.05 (*n* = 8). ^a^ significantly different vs. normal control, ^b^ significantly different vs. BLM-positive control, ^c^ significantly different vs. PFD alone, ^d^ significantly different vs. MET alone, ^e^ significantly different vs. BM-MSC group.

Interleukin-10 (IL-10), an anti-inflammatory cytokine, was assessed in the lung tissues of the experimental rats. The BLM-positive control group exhibited a significant reduction of 52.3% in IL-10 levels when compared to the normal control levels. The combined treatment group showed a 2.05-fold rise in IL-10 levels compared to the BLM-positive control, surpassing the results of the PFD, MET, and BM-MSC groups, which reported increases of 1.86-fold, 1.90-fold, and 1.91-fold, respectively. The IL-10 levels in the combined treatment group were statistically comparable to those of the normal control group (*p* > 0.05) (Figure 4A) (Table 2).

Tumor necrosis factor-alpha (TNF-α), an important pro-inflammatory cytokine, was also quantified in the lung tissue using ELISA. The BLM-positive control group showed a significant increase in TNF-α levels by 4.04-fold compared to the normal control. Treatment with PFD, MET, and BM-MSCs led to considerable reductions in TNF-α levels by 46.70%, 51.81%, and 54%, respectively, when compared to the BLM-positive control. However, the combination treatment achieved the most significant decrease, with TNF-α levels reduced by 69.07%. The TNF-α levels in the combined treatment group were statistically similar to those in the normal control group (*p* > 0.05) (Figure 4B) (Table 2).

Transforming growth factor-beta1 (TGF-β1), a key participant in the development of pulmonary fibrosis, was significantly elevated in the BLM-positive control group, demonstrating a 2.8-fold increase relative to the normal control (*p* < 0.05). Administration of PFD, MET, BM-MSCs, and the combination therapy resulted in notable reductions in TGF-β1 levels by 42.55%, 43.19%, 49.96%, and 53.02%, respectively, in comparison to the BLM-positive control (*p* < 0.05) (Figure 4C) (Table 2).

### 3.5. The Expression Levels of Col1α1 and MMP-9 in the Lung Tissue of the Experimental Rats

**Figure 5:**
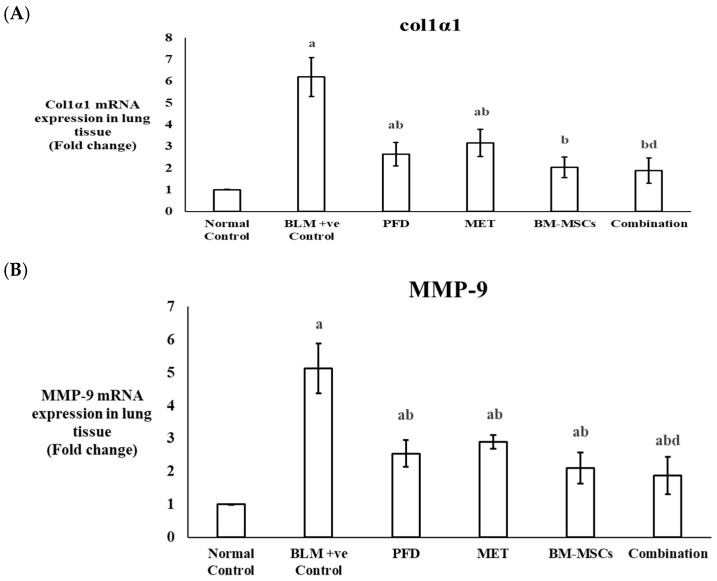
The expression levels of Col1α1 and MMP-9 in the lung tissue of the experimental rats. (**A**) Col1α1 and (**B**) MMP-9. Col1α1 = collagen type1 alpha1 chain; MMP-9 = metalloproteinase 9. PFD = pirfenidone; MET = metformin; BM-MSCs = bone marrow-derived mesenchymal stem cells. The parameters were detected by quantitative PCR. Data were expressed as mean ± SD. Data are expressed as mean ± SD. Data were analyzed by one-way ANOVA followed by Bonferroni’s post hoc test. Differences were considered significant at *p* < 0.05 (*n* = 8). ^a^ significantly different vs. normal control, ^b^ significantly different vs. BLM-positive control, ^d^ significantly different vs. MET alone.

Real-time PCR was utilized to assess the mRNA expression levels of *Col1α1* and *MMP-9* in lung tissue samples. The BLM-positive control group demonstrated a 6.13-fold increase in *Col1α1* expression compared to the normal control group (*p* < 0.05). Treatment with PFD, MET, BM-MSCs, or the combination therapy significantly decreased *Col1α1* expression levels by 57.25%, 49.03%, 67.09%, and 69.67%, respectively, when compared to the BLM-positive control group (*p* < 0.05). Notably, there was no significant difference in *Col1α1* expression observed between the BM-MSC group or the combined treatment group and the normal control group (*p* > 0.05) (Figure 5A) (Table 2).

The BLM-positive control group also showed a 5.13-fold increase in *MMP-9* expression levels compared to the normal control group (*p* < 0.05). Treatment with PFD, MET, or BM-MSCs alone significantly reduced *MMP-9* expression levels by 50.48%, 43.46%, and 59.06%, respectively. The combination treatment resulted in the largest reduction, lowering *MMP-9* expression by 63.35% compared to the BLM-positive control group (*p* < 0.05) (Figure 5B) (Table 2).

### 3.6. Histopathological Examination of Lung Tissue in Experimental Rats

**Figure 6:**
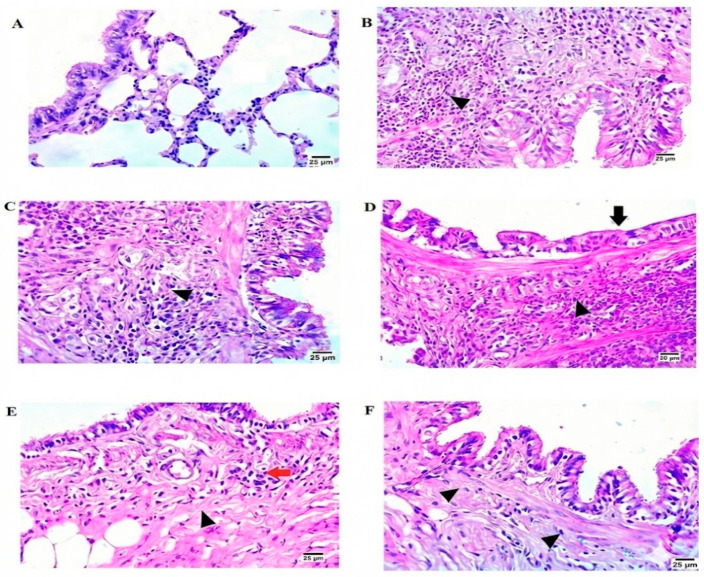
Histopathological examination (H&E) of lung tissue sections of the experimental rats. (**A**) Normal control group showing normal histological structure of the lung. (**B**) Bleomycin-positive control group showing heavy peribronchiolar mononuclear infiltration (black head). (**C**) Pirfenidone-treated group showing vacuolated epithelial lining, darky-stained nuclei and heavy mononuclear infiltration (black head). (**D**) Metformin-treated group showing minimal vacuolated cytoplasm in epithelial lining of bronchioles (arrow). Some cells had pycnotic nuclei. There was heavy mononuclear infiltration (black head). (**E**) BM-MSCs-treated group showing vacuolated cytoplasm and pycnotic nuclei of epithelial lining of bronchioles (black head). Bronchioles were surrounded by CT that has thick fibers and less infiltrating cells (red arrow). (**F**) Pirfenidone + metformin + BM-MSCs combination-treated group showing bronchioles surrounded by more CT fibers and less cells (black head). Epithelial lining was vacuolated with pycnotic nuclei. All images are of tissue H&E stained at magnification ×400. BM-MSCs = bone marrow mesenchymal stem cells.

Histopathological examination of lung tissue utilizing H&E staining indicated notable variations among the experimental groups. The normal control group displayed a typical lung structure with no signs of pathology (Figure 6A). Conversely, the BLM-positive control group exhibited severe peribronchiolar fibrosis along with a significant infiltration of mononuclear inflammatory cells (Figure 6B). The PFD-treated group showed evidence of perialveolar fibrosis, vacuolated epithelial lining, and darkly stained nuclei, alongside a substantial infiltration of mononuclear inflammatory cells (Figure 6C). In the MET-treated group, histological assessment indicated minimal vacuolation in the epithelial cytoplasm of bronchioles, with some cells presenting pycnotic nuclei, epithelial atrophy in the bronchioles, peribronchiolar fibrosis, and a high level of mononuclear inflammatory cell infiltration (Figure 6D). Treatment with BM-MSCs resulted in vacuolated cytoplasm and pycnotic nuclei in the bronchiolar epithelial lining as well as minimal infiltration of mononuclear inflammatory cells and reduced peribronchiolar fibrosis (Figure 6E). Importantly, the combined treatment group exhibited the most considerable improvement, featuring epithelial vacuolation of bronchiolar lining, pycnotic nuclei, and a very low presence of mononuclear inflammatory cell infiltration (Figure 6F).

**Figure 7:**
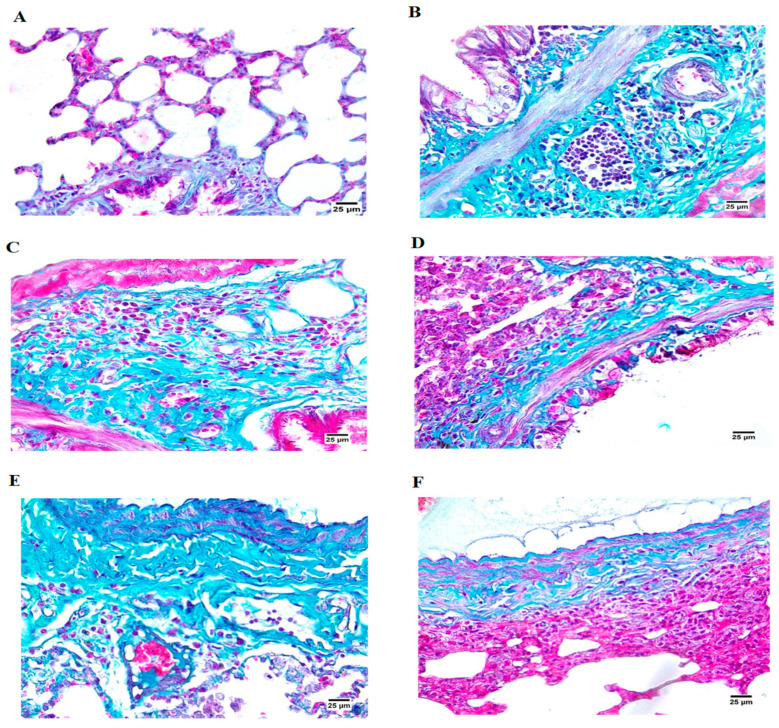
Histopathological examination (Masson’s trichrome) of lung tissue sections of the experimental rats. (**A**) Normal control group showing normal histological structure of the lung. (**B**) pulmonary fibrosis control group showing vacuolated epithelial lining of bronchioles and peribronchiolar thick connective tissue (CT) heavily infiltrated with mononuclear cells. (**C**) Pirfenidone-treated group showing thick CT around the bronchioles and separating the few alveoli. There was mononuclear infiltration. (**D**) Metformin-treated group showing degenerated epithelial lining (cytoplasm and nuclei). Bronchioles were surrounded by thick CT fibers and heavy infiltration. (**E**) BM-MSCs-treated group showing thick CT fibers. (**F**) Pirfenidone + metformin + BM-MSCs combination-treated group showing few collapsed lung alveoli separated by CT that has thick bundles of fibers and infiltrating cells. All images are at magnification of ×400. BM-MSCs = bone marrow mesenchymal stem cells.

Histopathological assessment of lung tissue using Masson’s trichrome staining similarly indicated significant differences among the experimental groups. The normal control group showcased an undamaged and regular lung structure (Figure 7A). In contrast, the BLM-administered group presented with vacuolated epithelial lining in bronchioles and thick connective tissue (CT) fibers surrounding the peribronchiolar region, along with severe infiltration of mononuclear cells (Figure 7B). PFD treatment demonstrated thick CT fibers encircling the bronchioles and separating a few alveoli, alongside mononuclear infiltration (Figure 7C). Rats receiving MET treatment showed degeneration of the epithelial lining (both cytoplasm and nuclei), with bronchioles enveloped by thick CT fibers and heavy infiltration (Figure 7D). Similarly, treatment with BM-MSCs and combination therapy revealed the presence of thick CT fibers, with the combination therapy displaying few collapsed lung alveoli and infiltrating cells (Figure 7E,F).

### 3.7. Semi-Quantitative Evaluation of Pulmonary Fibrosis Using the Ashcroft Scoring System (0–8 Scale)

Evaluation of lung fibrosis showed a significant increase in fibrosis score in BLM-positive control group (6.5 ± 0.53) compared to normal control group (0.38 ± 0.52 (*p* < 0.0001)). Meanwhile, treatment with pirfenidone significantly decreased fibrosis when compared to the BLM-positive control (4.63 ± 0.52). Furthermore, the treatment with metformin led to a moderate decrease in fibrosis score (5.75 ± 0.46) compared to the BLM group. The results for the group treated with the BM-MSCs showed a more significant antifibrotic effect (2.75 ± 0.46) with significant decrease in collagen deposition and lung architectural repair. However, the combination treatment group had the best recovery and the lowest fibrosis score (1.75 ± 0.46) when compared to the monotherapy groups. It indicated a nearly normal histological appearance. Overall, our results show that bleomycin induced substantial fibrosis in the lung, and all treatment measures, to a different extent, slowed the progression of fibrosis, with combination therapy showing the greatest antifibrotic effect.

## 4. Discussion

Pulmonary fibrosis represents a severe and progressive lung disorder characterized by the gradual loss of functional alveoli due to the replacement of normal tissue with fibrotic scar tissue [18]. This irreversible damage leads to a decline in pulmonary function, culminating in respiratory failure and often death [19]. At the cellular level, this condition involves complex interactions between oxidative stress, inflammation, apoptosis, and fibrogenesis, all contributing to the pathogenesis of the disease. In this study, we investigated the combined therapeutic effects of PFD, MET, and BM-MSCs on BLM-induced pulmonary fibrosis in rats. Our findings demonstrate that this multimodal therapy significantly mitigated oxidative stress, reduced inflammation, inhibited apoptosis, and suppressed fibrosis more effectively than monotherapy alone.

Apoptosis, a tightly regulated form of programmed cell death, plays a central role in the development of pulmonary fibrosis [20]. It involves the controlled disassembly of cells into apoptotic bodies, which are then phagocytosed without inducing an inflammatory response. However, in fibrotic diseases, excessive apoptosis of alveolar epithelial cells contributes to tissue damage, while reduced apoptosis in myofibroblasts allows for their persistence and continued deposition of extracellular matrix (ECM), promoting fibrosis [21]. Additionally, oxidative stress, defined as an imbalance between ROS production and antioxidant defenses, is a major driver of lung injury and fibrosis. The lungs are particularly vulnerable to oxidative damage due to continuous exposure to oxygen and environmental oxidants [22]. ROS activate inflammatory pathways, promote ECM accumulation, and trigger signaling cascades that lead to fibrotic remodeling.

In preclinical research, the intratracheal administration of BLM remains one of the most widely used models for studying pulmonary fibrosis [21]. BLM induces DNA damage via iron-dependent generation of free radicals, leading to lipid peroxidation, protein oxidation, and ultimately, cell death and fibrotic scarring. In our experimental model, BLM treatment caused significant increases in MDA and NO, markers of oxidative stress, alongside marked reductions in antioxidants such as GSH and SOD. These biochemical changes were accompanied by elevated levels of pro-inflammatory cytokines, including TNF-α and TGF-β1, and decreased anti-inflammatory IL-10. Histopathological examination further confirmed the presence of extensive collagen deposition, thickened interstitial septa, and inflammatory infiltration, consistent with established models of BLM-induced fibrosis [23,24].

Pirfenidone is a well-established antifibrotic agent with pleiotropic effects, including anti-inflammatory, antioxidant, and antifibrotic properties [25]. In our study, PFD significantly ameliorated BLM-induced oxidative stress by lowering MDA and NO levels while enhancing GSH and SOD activity. These results align with previous studies showing that PFD enhances antioxidant gene expression and reduces oxidative damage in animal models of lung injury [26,27]. Furthermore, it has also been shown that pirfenidone is able to increase Nrf2-mediated defenses against oxidants, which is a factor that has added to its protective effects against oxidative damage in fibrotic and other disease models. Nuclear factor erythroid 2-related factor 2 (Nrf2) is a master controller of cellular antioxidant responses, which induces transcription of phase-2 detoxifying and redox-balancing genes including heme oxygenase-1 (HO-1) and glutathione peroxidase-1 (Gpx1) when released by its inhibitory partner Bach1. Treatment with pirfenidone in experimental models of bleomycin-induced pulmonary fibrosis repressed Bach1, reversed the inhibitory effect of the profibrotic stimuli on the antioxidant system, and increased the expression of Nrf2 and its downstream effectors HO-1 and Gpx1, leading to a reduction in the reactive oxygen species and lipid peroxidation indicators, blocking inflammation and reducing fibrotic pathology. This Nrf2/Bach1 balance indicates that pirfenidone increases endogenous antioxidant ability through Nrf2 activation and facilitation of cytoprotective antioxidant biomarkers of oxidative stress-induced tissue damage underlying fibrotic pathogenesis [26]. Moreover, PFD increased the anti-apoptotic protein Bcl-2 and decreased the pro-apoptotic marker BAX, indicating its ability to modulate the intrinsic apoptotic pathway. It has also been claimed that pirfenidone has the ability to control intrinsic apoptotic pathways in restoring the balance between pro- and anti-apoptotic Bcl-2 family proteins, which is essential to preserve cell survival in response to fibrotic and injury stimuli. Treatment with pirfenidone in models of lung injury and emphysema had a significant effect of lowering the apoptotic index of the damaged cells and reversing stress-induced changes in apoptosis-related proteins, such as decreasing pro-apoptotic protein Bax and increasing anti-apoptotic protein Bcl-2, leading to a restored cell-surviving normal Bcl-2/Bax ratio and alleviating apoptosis through the mitochondrial pathway. This outcome of pirfenidone coincided with a parallel decrease in cleaved caspase-3 levels, which indicated inhibition of downstream executioner caspase activity and suggested that the protective effect of pirfenidone was due to inhibition of the endogenous mitochondrial apoptotic cascade and, thus, reinforces its cytoprotective and antifibrotic actions in the experimental disease models [28]. Consistent with earlier reports [6,29,30], PFD also suppressed TNF-α and TGF-β1 levels while elevating IL-10, demonstrating its immunomodulatory capacity. Alongside our findings, it was reported that pirfenidone also has antifibrotic and anti-inflammatory effects that are mainly observed to inhibit the signaling pathway of TGF-b/Smad. It inhibits phosphorylation of Smad2 and Smad3 mediated by TGF-β1 to suppress myofibroblast differentiation, extracellular matrix synthesis and epithelial–mesenchymal transition, which are involved in its antifibrotic effects [31]. Besides its impact on Smad signaling, pirfenidone has been shown to suppress the nuclear factor-κB pathway which is considered as a major mediator of inflammatory gene expression. PFD treatment inhibits NF-κB activation and subsequent production of pro-inflammatory cytokines in various disease models, indicating that NF-κB signaling inhibition also contributes to its anti-inflammatory and tissue-protective effects in fibrotic diseases [32].

At the molecular level, PFD downregulated the expression of *Col1α1* and *MMP-9*, two key genes involved in ECM deposition and remodeling. In line with our results, the ability of pirfenidone to suppress collagen gene expression has been extensively reported to reduce overproduction of extracellular matrix (ECM) which defines fibrotic diseases. Mechanistic studies have shown that pirfenidone inhibits transforming growth factor-1 (TGF-1)-stimulated transcriptional activation of collagen genes especially *Col1α1* and *Col3α1* in lung fibroblasts as well as alveolar epithelial cells. Pirfenidone dramatically lowers collagen type I mRNA expression and TGF-β-mediated profibrotic signaling in human lung fibroblasts of patients with idiopathic pulmonary fibrosis (IPF), suggesting that it has a direct effect on transcriptional regulation of collagen synthesis, thereby playing a significant role in its antifibrotic therapeutic efficacy [33]. Histological analysis revealed reduced collagen accumulation in the interalveolar septa and bronchiolar walls, corroborating the molecular data. These findings support the notion that PFD exerts its therapeutic effects through multiple mechanisms, including suppression of TGF-β signaling, inhibition of ROS generation, and modulation of inflammatory cytokine release [6,15,34,35].

Metformin, a first-line antidiabetic drug, has emerged as a promising antifibrotic agent due to its ability to activate AMP-activated protein kinase (AMPK) and inhibit mammalian target of rapamycin (mTOR) signaling [16]. The multidimensional impacts of metformin on cellular energy homeostasis may probably correlate with the stimulation of the adenosine monophosphate-activated protein kinase (AMPK) involved in the energy regulation of cells. The metformin inhibits the activity of respiratory complex I in mitochondria, raising intracellular AMP/ATP, which causes the phosphorylation and activation of AMPK. Some of the mechanisms by which the activated AMPK can restore metabolic homeostasis include the activation of catabolic pathways, the inhibition of anabolic processes, the regulation of cellular proliferation, inflammation, and stress responses. Notably, AMPK activation is linked closely to suppression of the mammalian target of rapamycin complex 1 (mTORC1) signal by phosphorylation of upstream regulators including TSC2 and Raptor, resulting in the inactivation of downstream effectors like p70S6 kinase and 4E-BP1. This suppresses protein synthesis, cell growth, and proliferative signaling but increases autophagy. The AMPK/mTOR axis is modulated to support metabolic, anti-proliferative, and cytoprotective actions of metformin in various pathological mechanisms that are relevant to different fields of therapeutic application in addition to managing diabetes [36,37]. In our study, MET treatment significantly reduced oxidative stress markers and restored antioxidant enzyme levels, consistent with previous findings in rodent models of pulmonary fibrosis [16,38]. Metformin can inhibit the production of reactive oxygen species (ROS), which is important in the alleviation of oxidative stress and cellular damage in various models. Metformin significantly suppressed the production of ROS in response to inflammatory stimuli in human macrophages, which was accompanied by the activation of AMP-activated protein kinase (AMPK), indicating that the effect of metformin on the inhibition of ROS is mediated by AMPK-mediated regulation of oxidative stress and NADPH oxidase activity [39]. According to another recent study in glucose-induced oxidative injury, it was observed that metformin can induce the Nrf2/HO-antioxidant pathway, leading to reduced intracellular ROS and defense against oxidative stress caused by high-glucose levels [40]. The other consequence of MET was anti-apoptotic that increased the expression of Bcl-2 and decreased that of BAX suggesting that MET could preserve cell viability in stressful conditions. On the mechanistic level, MET suppressed the expression of pro-inflammatory cytokines, such as TNF-αand TGF-β, and augmented IL-10 and thereby restored the nitrogen balance within the inflammatory environment. MET has also been reported to have anti-inflammatory activity [41,42]. It has been demonstrated that metformin has prominent inhibitory effects on Smad2/3 phosphorylation and pro-inflammatory cytokine release due to various interlocking mechanisms related to the regulation of critical signaling pathways. Simultaneously, metformin inhibits the synthesis of pro-inflammatory cytokines like TNF-α, IL-6 and IL-1 β by regulating inflammatory signaling pathways, the most prominent of which involves the activation of AMP-activated protein kinase (AMPK), which in turn suppresses the activation of nuclear factor-_K_B (NF-_K_B), one of the main transcription factors that regulate the expression of cytokine genes, reducing the phosphorylation and activation of the NF-_K_B subunits and its activators [43,44].

Gene expression was found to be significantly suppressed by MET using *Col1α1* and MMP-9 which was supported by histological data on the decrease in collagen deposition and inflammatory infiltration. These data confirm earlier studies [42,45] and suggest that MET also suppresses fibrosis by targeting oxidative stress and inflammation as well as directing ECM turnover and fibroblasts activation.

BM-MSCs have received significant interest due to their regenerative and immunomodulatory capabilities in the therapy of fibrotic disease [8,9,10]. BM-MSC administration resulted in serious changes in oxidative stress parameters in our research such as lowering the MDA and NO levels and enhancing GSH and SOD activity, confirming their well-documented potent antioxidant properties [46,47]. Recently in preclinical research on pulmonary fibrosis, bone BM-MSCs have been discovered to reduce oxidative stress and enhance the activity of antioxidant enzymes in fibrotic lung tissues through well-defined molecular pathways. One of such mechanisms is the activation of nuclear factor erythroid 2-related factor 2 (Nrf2) signaling pathway, which is a master of regulating cellular redox balance, transcription of antioxidants and detoxification enzymes including heme oxygenase-1 (HO-1), superoxide dismutase, glutathione peroxidase and NADPH quinone oxidoreductase 1 (NQO1), which improves the endogenous defenses of the antioxidants [48]. These effects were associated with enhanced expression of Bcl-2 and reduced BAX levels, highlighting the previously reported anti-apoptotic actions of BM-MSCs in lung tissue [49,50]. Experimental pulmonary fibrosis models have demonstrated that bone BM-MSCs and their extracellular vesicles (e.g., MSC-derived exosomes) can inhibit pathological epithelial apoptosis by regulating the ratio of pro-apoptotic and anti-apoptotic proteins. The exosome secreted by MSCs has been shown to significantly reduce the upregulation of pro-apoptotic Bax gene expression and the downregulation of anti-apoptotic Bcl-2 in a bleomycin-induced fibrosis model in vitro and in vivo, which is associated with a decrease in apoptotic alveolar epithelial cells and fibrotic damage. This anti-apoptotic effect is mediated by the inhibition of endoplasmic reticulum (ER) stress pathways that would induce epithelial cell death in fibrotic lungs because exosome treatment by MSCs reduced markers of ER stress along with its effects on Bax/Bcl-2 and apoptotic indices in bleomycin-treated A549 cells and in lung tissues. This results in enhanced structural integrity and fibrosis in these preclinical models with the subsequent rise in epithelial cell survival [51].

BM-MSCs also exerted potent anti-inflammatory effects, as evidenced by reduced TNF-α and TGF-β1 levels and increased IL-10 concentrations. BM-MSCs mediate antifibrotic effects in experimental models of pulmonary fibrosis by their paracrine release of regulatory mediators but not direct cell engraftment. Their secretomes are augmented in antifibrotic and anti-inflammatory actions, such as hepatocyte growth factor (HGF), prostaglandin E 2 (PGE 2), and interleukin-10 (IL-10), which all suppress TGF-β1 signaling, inhibit collagen deposition, and modulate tissue microenvironment. Also, BM-MSC paracrine stimulates immunomodulation and IL-10 upregulation and downregulation of pro-inflammatory cytokines, suppressing chronic inflammation, which is a major cause of fibrosis, promoting fibrosis recovery and tissue repair. These mechanisms explain the importance of MSC-secretory factors to enhance antifibrotic actions in lung injury models [52]. Histopathological evaluation demonstrated a marked reduction in peribronchiolar fibrosis and inflammatory cell infiltration, consistent with previous studies showing that MSCs can suppress macrophage activation, reduce myofibroblast proliferation, and enhance collagen degradation via MMP-9 upregulation [15,53]. Furthermore, BM-MSCs significantly downregulated *Col1α1* and *MMP-9* expression, reinforcing their role in modulating fibrogenic pathways. BM-MSCs can alleviate pulmonary fibrosis by suppressing fibroblast activation to myofibroblasts and regulating innate immunity by polarizing macrophages into the anti-inflammatory M2 phenotype. This effect is mediated through paracrine signaling, including chemokines that stimulate M2 differentiation (CD206) and inhibit pro-inflammatory M1 macrophages, resulting in reduced release of inflammatory cytokines and inhibiting fibrogenic signaling. As a result, BM-MSC therapy decreases extracellular matrix deposition, collagen deposition, and fibrotic marker expression and promotes tissue healing, which demonstrates dual antifibrotic and immunoregulatory effects of MSCs in fibrotic lung disease [54].

The most notable finding of this study is the superior therapeutic efficacy of the combination therapy consisting of PFD, MET, and BM-MSCs compared to individual treatments. The combination therapy, which appears additive/multimodal rather than synergistic, was performed to produce greater normalization of oxidative stress and inflammatory markers.

Significantly, MSC phenotype was confirmed following culture expansion, relieving concerns about phenotypic drift. This multimodal approach resulted in the most pronounced reductions in oxidative stress markers and the greatest enhancement of antioxidant defenses. Apoptotic regulation was also maximally improved, with the highest Bcl-2 and lowest BAX levels observed in the combination group. Importantly, the combination therapy achieved the most significant suppression of pro-inflammatory cytokines (TNF-α and TGF-β1) and the highest elevation of anti-inflammatory cytokine IL-10, underscoring its broad immunomodulatory impact.

At the molecular level, the combination therapy induced the most substantial downregulation of *Col1α1* and *MMP-9*, indicating effective inhibition of fibrogenesis and ECM remodeling. Histologically, the combination-treated group showed minimal collagen deposition, preserved lung architecture, and negligible inflammatory infiltration, closely resembling the normal control group. These findings suggest that the synergistic interactions among PFD, MET, and BM-MSCs targets multiple pathological pathways simultaneously, offering a comprehensive strategy for reversing established fibrosis.

Supporting our results, recent studies have shown that combining PFD and MET enhances antifibrotic outcomes in pulmonary fibrosis models [24,55]. Similarly, co-culturing adipose-derived stem cells with MET has been reported to suppress inflammation and fibrotic gene expression more effectively than either agent alone [56]. Moreover, low-dose PFD combined with human umbilical mesenchymal stem cells (HUMSCs) has been shown to produce greater antifibrotic effects than high-dose PFD alone, potentially reducing side effects and improving patient compliance [55].

This study provides compelling evidence for the therapeutic potential of combining PFD, MET, and BM-MSCs in treating pulmonary fibrosis. Given that current therapies for idiopathic pulmonary fibrosis (IPF) primarily slow disease progression rather than reverse existing damage, the identification of novel, synergistic treatment strategies are of critical importance. Our findings suggest that targeting oxidative stress, inflammation, apoptosis, and fibrosis simultaneously may offer a more robust therapeutic effect than monotherapies.

However, several limitations must be acknowledged. First, the study was conducted in a rat model of BLM-induced fibrosis, which may not fully recapitulate the complexity of human IPF. Second, long-term safety and efficacy assessments are needed before clinical translation. Third, formal synergy analysis was not performed and the precise mechanisms underlying the synergistic effects of the combination therapy require further elucidation, particularly regarding paracrine signaling and cellular interactions between BM-MSCs and resident lung cells. Fourth, although the study showed notable anti-inflammatory and antifibrotic outcomes, it did not investigate the underlying molecular mechanisms by analyzing key signaling pathways like AMPK/mTOR, NF-κB, Nrf2, or TGF-β/Smad. This would have contributed to deeper mechanistic understanding of the observed therapeutic synergy.

Despite these challenges, our work opens new avenues for developing integrated therapeutic approaches for pulmonary fibrosis. Future studies should focus on optimizing dosing regimens, exploring delivery methods, and evaluating the safety profile of this combination in larger animal models and eventually in clinical trials.

## 5. Conclusions

According to this study, the major features of bleomycin-induced pulmonary fibrosis are increased oxidative stress, inflammatory response, apoptotic imbalance, and an excess of extracellular matrix deposition in lung tissue. Treatment with pirfenidone, metformin or BM-MSCs alone improved these pathogenic abnormalities to some extent. The combination therapy had the largest effect, demonstrated by the restoration of antioxidant defenses, inhibition of pro-inflammatory mediators, modulation of apoptotic markers and a significant decrease in the expression of fibrogenic genes and collagen deposits. The histological findings of the combination group showed further proof for the reduction in lung fibrosis and architectural damage. All of these findings suggest that combination therapy treatment of multiple pathologic pathways may result in greater antifibrotic effects in experimental lung fibrosis.

## Figures and Tables

**Table 1:** Primers and annealing temperatures used in real-time PCR.

Gene	Primers	Annealing Temperature
*MMP-9*	Forward: 5′-TCGAAGGCGACCTCAAGTG-3′ Reverse: 5′-TTCGGTGTAGCTTTGGATCCA-3′ [17].	53 °C
*Col1α1*	Forward: 5′-TTCCTGCCTCAGCCACCTCA-3′ Reverse: 5′-GAACCTTCGCTTCCATACTCG-3′ [15].	52 °C
*β-actin*	Forward: 5′-GACCTTCAACACCCCAGCCA-3′ Reverse: 5′-GTCACGCACGATTTCCCTCTC-3′ [17].	55 °C

**Table 2:** Levels of oxidative stress, apoptotic, inflammatory markers and expression levels of Col1α1 and MMP-9 in the lung tissue of the experimental rats (mean ± SD).

	Normal Control	BLM + ve Control	PFD	MET	BM-MSCs	Combination	*p*-Value
MDA (mmol/g tissue)	35.35 ± 2.03	135.2 ± 7.27 a	74.16 ± 8.16 ab	73.34 ± 3.93 ab	64.53 ± 10.37 abc	51.44 ± 7.55 abcde	<0.0001
NO (mmol/g tissue)	15.79 ± 3.64	83.65 ± 7.67 a	49.65 ± 4.89 ab	37.98 ± 4.73 abc	30.01 ± 6.09 abc	24.91 ± 5.53 abcd	<0.0001
GSH (mmol/g tissue)	127.4 ± 11.23	56.59 ± 8.06 a	99.74 ± 6.01 ab	108.4 ± 5.99 ab	104.8 ± 9.87 ab	123.9 ± 5.10 bcde	<0.0001
SOD (mmol/g tissue)	90.40 ± 4.89	29.96 ± 5.97 a	67.41 ± 9.38 ab	69.54 ± 6.16 ab	69.25 ± 9.08 ab	81.75 ± 6.13 bcde	<0.0001
Bcl-2 (mmol/g tissue)	333.5 ± 12.73	114.7 ± 3.85 a	296.1 ± 18.89 ab	306.5 ± 14.14 ab	309.9 ± 11.25 ab	326.9 ± 8.91 bcde	<0.0001
BAX (mmol/g tissue)	119.1 ± 8.77	269.7 ± 9.33 a	177.7 ± 16.13 ab	166.2 ± 15.33 ab	154.2 ± 15.05 abc	127.4 ± 6.89 bcde	<0.0001
IL-10 (mmol/g tissue)	445.7 ± 10.30	212.4 ± 7.27 a	399.6 ± 8.46 ab	407.1 ± 8.84 ab	410.8 ± 11.42 ab	434.8 ± 10.14 bcde	<0.0001
TNF-α (mmol/g tissue)	46.29 ± 5.38	187.4 ± 4.71 a	95.90 ± 10.41 ab	85.95 ± 9.33 ab	81.46 ± 10.30 abc	57.94 ± 10.10 abcde	<0.0001
TGF-β1 (mmol/g tissue)	69.88 ± 5.92	196.5 ± 14.09 a	110.7 ± 5.67 ab	107.1 ± 9.77 ab	98.31 ± 11.06 ab	94.58 ± 10.12 abcd	<0.0001
Col1α1 (Fold change)	1.006 ± 0.01	6.775 ± 2.22 a	2.835 ± 0.57 ab	3.158 ± 0.63 ab	2.039 ± 0.47 b	1.879 ± 0.58 bd	<0.0001
MMP-9 (Fold change)	1.004 ± 0.01	5.188 ± 0.96 a	2.68 ± 0.42 ab	2.73 ± 0.36 ab	2.10 ± 0.48 ab	1.875 ± 0.57 abd	<0.0001

Data were analyzed by one-way ANOVA followed by Bonferroni’s post hoc test. Differences were considered significant at *p* < 0.05 (*n* = 8). (a) significantly different vs. normal control, (b) significantly different vs. BLM-positive control, (c) significantly different vs. PFD alone, (d) significantly different vs. MET alone, (e) significantly different vs. BM-MSC group.

## Data Availability

Data is available within the article.

## Abbreviations

The following abbreviations are used in this manuscript:

PFD	Pirfenidone
MET	Metformin
BM-MSCs	Bone Marrow-Derived Mesenchymal Stem Cells
BLM	Bleomycin
H&E	Hematoxylin and Eosin
BAX	Bcl-2 Associated X Protein
BcL-2	B-Cell Lymphoma-2
IL-10	Interleukin-10
TNF-α	Tumor Necrosis Factor-Alpha
TGF-β1	Transforming Growth Factor-Beta 1
MMP-9	Matrix Metalloproteinase-9
Col1α1	Collagen Type I Alpha 1
IL-1	Interleukin-1
IL-13	Interleukin-13
AMP	Adenosine Monophosphate
AMPK	AMP-Activated Protein Kinase
ROS	Reactive Oxygen Species
PBS	Phosphate-Buffered Saline
CO_2_	Carbon Dioxide
DMEM	Dulbecco’s Modified Eagle Medium
FBS	Fetal Bovine Serum
PE	Phycoerythrin
FITC	Fluorescein Isothiocyanate
ELISA	Enzyme-linked immunosorbent assay
EDTA	Ethylenediaminetetraacetic acid
CD90	Cluster of Differentiation 90
CD105	Cluster of Differentiation 105
CMC	carboxymethylcellulose
RPM	Revolutions Per Minute
COX-2	Cyclooxygenase-2
NF-κB	Nuclear Factor Kappa-B
CAT	Catalase
MDA	Malondialdehyde
GSH	Reduced Glutathione
SOD	Superoxide Dismutase
NO	Nitric Oxide
cDNA	Complementary Deoxyribonucleic Acid
PCR	Polymerase Chain Reaction
SD	Standard Deviation
CT	Connective tissue
ANOVA	Analysis of Variance
ECM	Extracellular Matrix
mTOR	Mammalian Target of Rapamycin
Nrf2	Nuclear factor erythroid 2–related factor 2
HUMSCs	Human Umbilical Mesenchymal Stem Cells
IPF	Idiopathic Pulmonary Fibrosis
TIMP-1	Tissue Inhibitor of Metalloproteinase-1

## References

[1] Vats A., Chaturvedi P. (2023). The Regenerative Power of Stem Cells: Treating Bleomycin-Induced Lung Fibrosis. Stem Cells Cloning Adv. Appl..

[2] Allawzi A., Elajaili H., Redente E.F., Nozik-Grayck E. (2018). Oxidative Toxicology of Bleomycin: Role of the Extracellular Redox Environment. Curr. Opin. Toxicol..

[3] Chen J., Ghorai M.K., Kenney G., Stubbe J.A. (2008). Mechanistic studies on bleomycin-mediated DNA damage: Multiple binding modes can result in double-stranded DNA cleavage. Nucleic Acids Res..

[4] Zhou Z., Kandhare A.D., Kandhare A.A., Bodhankar S.L. (2019). Hesperidin ameliorates bleomycin-induced experimental pulmonary fibrosis via inhibition of TGF-β1/Smad3/AMPK and IκBα/NF-κB pathways. EXCLI J..

[5] Jang S., Ryu S.M., Lee J., Lee H., Hong S.-H., Ha K.-S., Park W.S., Han E.-T., Yang S.-R. (2018). Bleomycin Inhibits Proliferation via Schlafen-Mediated Cell Cycle Arrest in Mouse Alveolar Epithelial Cells. Tuberc. Respir. Dis..

[6] Antar S.A., Saleh M.A., Al-Karmalawy A.A. (2022). Investigating the possible mechanisms of pirfenidone to be targeted as a promising anti-inflammatory, anti-fibrotic, anti-oxidant, anti-apoptotic, anti-tumor, and/or anti-SARS-CoV-2. Life Sci..

[7] Wu M., Xu H., Liu J., Tan X., Wan S., Guo M., Long Y., Xu Y. (2021). Metformin and Fibrosis: A Review of Existing Evidence and Mechanisms. J. Diabetes Res..

[8] Zhang E., Yang Y., Zhang J., Ding G., Chen S., Peng C., Lavin M.F., Yeo A.J., Du Z., Shao H. (2019). Efficacy of bone marrow mesenchymal stem cell transplantation in animal models of pulmonary fibrosis after exposure to bleomycin: A meta-analysis. Exp. Ther. Med..

[9] Jovic D., Yu Y., Wang D., Wang K., Li H., Xu F., Liu C., Liu J., Luo Y. (2022). A Brief Overview of Global Trends in MSC-Based Cell Therapy. Stem Cell Rev. Rep..

[10] Yang Y., Chen Y., Liu Y., Ning Z., Zhang Z., Zhang Y., Xu K., Zhang L. (2023). Mesenchymal stem cells and pulmonary fibrosis: A bibliometric and visualization analysis of literature published between 2002 and 2021. Front. Pharmacol..

[11] Khalil M.R., El-Demerdash R.S., Elminshawy H.H., Mehanna E.T., Mesbah N.M., Abo-Elmatty D.M. (2021). Therapeutic effect of bone marrow mesenchymal stem cells in a rat model of carbon tetrachloride induced liver fibrosis. Biomed. J..

[12] Dominici M., Le Blanc K., Mueller I., Slaper-Cortenbach I., Marini F.C., Krause D.S., Deans R.J., Keating A., Prockop D.J., Horwitz E.M. (2006). Minimal criteria for defining multipotent mesenchymal stromal cells. The International Society for Cellular Therapy position statement. Cytotherapy.

[13] Bourin P., Bunnell B.A., Casteilla L., Dominici M., Katz A.J., March K.L., Redl H., Rubin J.P., Yoshimura K., Gimble J.M. (2013). Stromal cells from the adipose tissue-derived stromal vascular fraction and culture expanded adipose tissue-derived stromal/stem cells: A joint statement of the International Federation for Adipose Therapeutics and Science (IFATS) and the International Society for Cellular Therapy (ISCT). Cytotherapy.

[14] Wang H., Xu H., Lyu W., Xu Q., Fan S., Chen H., Wang D., Chen J., Dai J. (2022). KLF4 regulates TERT expression in alveolar epithelial cells in pulmonary fibrosis. Cell Death Dis..

[15] Chu K.A., Yeh C.C., Kuo F.H., Lin W.R., Hsu C.W., Chen T.H., Fu Y.S. (2020). Comparison of reversal of rat pulmonary fibrosis of nintedanib, pirfenidone, and human umbilical mesenchymal stem cells from Wharton’s jelly. Stem Cell Res. Ther..

[16] Li S.X., Li C., Pang X.R., Zhang J., Yu G.C., Yeo A.J., Lavin M.F., Shao H., Jia Q., Peng C. (2021). Metformin Attenuates Silica-Induced Pulmonary Fibrosis by Activating Autophagy via the AMPK-mTOR Signaling Pathway. Front. Pharmacol..

[17] Chen G., Ge D., Zhu B., Shi H., Ma Q. (2020). Upregulation of matrix metalloproteinase 9 (MMP9)/tissue inhibitor of metalloproteinase 1 (TIMP1) and MMP2/TIMP2 ratios may be involved in lipopolysaccharide-induced acute lung injury. J. Int. Med. Res..

[18] Savin I.A., Zenkova M.A., Sen’kova A.V. (2022). Pulmonary Fibrosis as a Result of Acute Lung Inflammation: Molecular Mechanisms, Relevant In Vivo Models, Prognostic and Therapeutic Approaches. Int. J. Mol. Sci..

[19] Raghu G., Remy-Jardin M., Myers J.L., Richeldi L., Ryerson C.J., Lederer D.J., Behr J., Cottin V., Danoff S.K., Morell F. (2018). Diagnosis of Idiopathic Pulmonary Fibrosis. An Official ATS/ERS/JRS/ALAT Clinical Practice Guideline. Am. J. Respir. Crit. Care Med..

[20] Yan N., Shi Y. (2005). Mechanisms of apoptosis through structural biology. Annu. Rev. Cell Dev. Biol..

[21] Safaeian L., Jafarian-Dehkordi A., Rabbani M., Sadeghi H.M., Afshar-Moghaddam N., Sarahroodi S. (2013). Comparison of bleomycin-induced pulmonary apoptosis between NMRI mice and C57BL/6 mice. Res. Pharm. Sci..

[22] Milenkovic M.R., Klisic A., Ceriman V., Stevuljevic J.K., Vujovic K.S., Mirkov D., Gajic M., Ilic B., Dimic N., Samardzic N. (2022). Oxidative stress and inflammation parameters-novel biomarkers for idiopathic pulmonary fibrosis. Eur. Rev. Med. Pharmacol. Sci..

[23] Elhady S.S., Goda M.S., Mehanna E.T., Elfaky M.A., Koshak A.E., Noor A.O., Bogari H.A., Malatani R.T., Abdelhameed R.F.A., Wahba A.S. (2022). Meleagrin Isolated from the Red Sea Fungus Penicillium chrysogenum Protects against Bleomycin-Induced Pulmonary Fibrosis in Mice. Biomedicines.

[24] Liu N., Song Y., Liu T., Wang H., Yu N., Ma H. (2024). Metformin enhanced the effect of pirfenidone on pulmonary fibrosis in mice. Clin. Respir. J..

[25] Maher T.M., Strek M.E. (2019). Antifibrotic therapy for idiopathic pulmonary fibrosis: Time to treat. Respir. Res..

[26] Liu Y., Lu F., Kang L., Wang Z., Wang Y. (2017). Pirfenidone attenuates bleomycin-induced pulmonary fibrosis in mice by regulating Nrf2/Bach1 equilibrium. BMC Pulm. Med..

[27] Bassiouny H.S., Salama N.M., El-Dean Habib A.S., Abdel-Hameed A.M. (2023). Histological Evaluation of The Possible Therapeutic Effect of Pirfenidone on Bleomycin-Induced Pulmonary Fibrosis in Adult Male Albino Rats. Egypt. J. Histol..

[28] Ma Y., Liu X., Luo L., Li H., Zeng Z., Chen Y. (2022). Effect of pirfenidone protecting against cigarette smoke extract induced apoptosis. Tob. Induc. Dis..

[29] Guo J., Yang Z., Jia Q., Bo C., Shao H., Zhang Z. (2019). Pirfenidone inhibits epithelial-mesenchymal transition and pulmonary fibrosis in the rat silicosis model. Toxicol. Lett..

[30] Torre A., Martínez-Sánchez F.D., Narvaez-Chávez S.M., Herrera-Islas M.A., Aguilar-Salinas C.A., Córdova-Gallardo J. (2024). Pirfenidone use in fibrotic diseases: What do we know so far?. Immun. Inflamm. Dis..

[31] Im C.Y., Kim S.H., Song K.H., Ryu M.O., Youn H.Y., Seo K.W. (2024). Pirfenidone inhibits TGF-β1-induced fibrosis via downregulation of Smad and ERK pathway in MDCK cells. Vet. Res. Commun..

[32] Zhang Z., Song J., Kwon S.H., Wang Z., Park S.G., Piao X., Ryu J.H., Kim N., Kim O.S., Kim S.H. (2023). Pirfenidone Inhibits Alveolar Bone Loss in Ligature-Induced Periodontitis by Suppressing the NF-κB Signaling Pathway in Mice. Int. J. Mol. Sci..

[33] Conte E., Gili E., Fagone E., Fruciano M., Iemmolo M., Vancheri C. (2014). Effect of pirfenidone on proliferation, TGF-β-induced myofibroblast differentiation and fibrogenic activity of primary human lung fibroblasts. Eur. J. Pharm. Sci..

[34] Hasdemir P.S., Ozkut M., Guvenal T., Uner M.A., Calik E., Koltan S.O., Koyuncu F.M., Ozbilgin K. (2017). Effect of Pirfenidone on Vascular Proliferation, Inflammation and Fibrosis in an Abdominal Adhesion Rat Model. J. Investig. Surg..

[35] Pourgholamhossein F., Rasooli R., Pournamdari M., Pourgholi L., Samareh-Fekri M., Ghazi-Khansari M., Iranpour M., Poursalehi H.-R., Heidari M.-R., Mandegary A. (2018). Pirfenidone protects against paraquat-induced lung injury and fibrosis in mice by modulation of inflammation, oxidative stress, and gene expression. Food Chem. Toxicol..

[36] Goel S., Singh R., Singh V., Singh H., Kumari P., Chopra H., Sharma R., Nepovimova E., Valis M., Kuca K. (2022). Metformin: Activation of 5′ AMP-activated protein kinase and its emerging potential beyond anti-hyperglycemic action. Front. Genet..

[37] Ma M., Pan Y., Zhang Y., Yang M., Xi Y., Lin B., Hao W., Liu J., Wu L., Liu Y. (2023). Metformin combined with rapamycin ameliorates podocyte injury in idiopathic membranous nephropathy through the AMPK/mTOR signaling pathway. J. Cell Commun. Signal..

[38] Gamad N., Malik S., Suchal K., Vasisht S., Tomar A., Arava S., Arya D.S., Bhatia J. (2018). Metformin alleviates bleomycin-induced pulmonary fibrosis in rats: Pharmacological effects and molecular mechanisms. Biomed. Pharmacother..

[39] Nassif R.M., Chalhoub E., Chedid P., Hurtado-Nedelec M., Raya E., Dang P.M.-C., Marie J.-C., El-Benna J. (2022). Metformin Inhibits ROS Production by Human M2 Macrophages via the Activation of AMPK. Biomedicines.

[40] Chen B., He Q., Yang J., Pan Z., Xiao J., Chen W., Chi W., Li M., Li S., Zeng J. (2023). Metformin suppresses Oxidative Stress induced by High Glucose via Activation of the Nrf2/HO-1 Signaling Pathway in Type 2 Diabetic Osteoporosis. Life Sci..

[41] Yan H., Zhou H.F., Hu Y., Pham C.T. (2015). Suppression of experimental arthritis through AMP-activated protein kinase activation and autophagy modulation. J. Rheum. Dis. Treat..

[42] Wu X., Xiao X., Chen X., Yang M., Hu Z., Shuai S., Fu Q., Yang H., Du Q. (2022). Effectiveness and mechanism of metformin in animal models of pulmonary fibrosis: A preclinical systematic review and meta-analysis. Front. Pharmacol..

[43] Lin H., Ao H., Guo G., Liu M. (2023). The Role and Mechanism of Metformin in Inflammatory Diseases. J. Inflamm. Res..

[44] Lin H., Li N., He H., Ying Y., Sunkara S., Luo L., Lv N., Huang D., Luo Z. (2015). AMPK Inhibits the Stimulatory Effects of TGF-β on Smad2/3 Activity, Cell Migration, and Epithelial-to-Mesenchymal Transition. Mol. Pharmacol..

[45] Xiao H., Huang X., Wang S., Liu Z., Dong R., Song D., Zhang X., Dai H. (2020). Metformin ameliorates bleomycin-induced pulmonary fibrosis in mice by suppressing IGF-1. Am. J. Transl. Res..

[46] Boughdady W., Hegab Z., Mohamed D. (2023). Therapeutic Effect of Bone Marrow Derived Mesenchymal Stem Cells Versus Platelet Rich Plasma on Amiodarone Induced Lung Fibrosis in Adult Male Albino Rat: Histological Study. Egypt. J. Histol..

[47] Adel A., Abdul-Hamid M., Abdel-Kawi S.H., Mallasiy L.O., Abd El-Gawaad N.S., Ahmed O.M. (2023). Bone marrow-derived mesenchymal stem cells abate CCl4-induced lung damage via their modulatory effects on inflammation, oxidative stress and apoptosis. Eur. Rev. Med. Pharmacol. Sci..

[48] Su H., Wang Z., Zhou L., Liu D., Zhang N. (2024). Regulation of the Nrf2/HO-1 axis by mesenchymal stem cells-derived extracellular vesicles: Implications for disease treatment. Front. Cell Dev. Biol..

[49] Chen X., Wu Y., Wang Y., Chen L., Zheng W., Zhou S., Xu H., Li Y., Yuan L., Xiang C. (2020). Human menstrual blood-derived stem cells mitigate bleomycin-induced pulmonary fibrosis through anti-apoptosis and anti-inflammatory effects. Stem Cell Res. Ther..

[50] Deng M., He J., Hao C., Guo Y., Si H., Deng X., Zhang C., Li S., Yao S., Ren W. (2023). Effect of Exosomes Derived from Bone Marrow Mesenchymal Stem Cells on Programmed Cell Death in Blast-Induced Lung Injury in Rats. Shock.

[51] Luo R., Wei Y., Wang L., Chen P., Lou D., Tian W. (2025). Targeting ER stress-mediated apoptosis by MSC-derived exosomes: A novel therapeutic strategy against pulmonary fibrosis. Regen. Ther..

[52] Chen Y., Liu X., Tong Z. (2023). Mesenchymal Stem Cells in Radiation-Induced Pulmonary Fibrosis: Future Prospects. Cells.

[53] Zhao X., Wu J., Yuan R., Li Y., Yang Q., Wu B., Zhai X., Wang J., Magalon J., Sabatier F. (2023). Adipose-derived mesenchymal stem cell therapy for reverse bleomycin-induced experimental pulmonary fibrosis. Sci. Rep..

[54] Hazrati A., Mirarefin S.M.J., Malekpour K., Rahimi A., Khosrojerdi A., Rasouli A., Akrami S., Soudi S. (2024). Mesenchymal stem cell application in pulmonary disease treatment with emphasis on their interaction with lung-resident immune cells. Front. Immunol..

[55] Wu X., Gou H., Zhou O., Qiu H., Liu H., Fu Z., Chen L. (2022). Human umbilical cord mesenchymal stem cells combined with pirfenidone upregulates the expression of RGS2 in the pulmonary fibrosis in mice. Respir. Res..

[56] Malekzadeh H., Surucu Y., Chinnapaka S., Yang K.S., Arellano J.A., Samadi Y., Epperly M.W., Greenberger J.S., Rubin J.P., Ejaz A. (2024). Metformin and adipose-derived stem cell combination therapy alleviates radiation-induced skin fibrosis in mice. Stem Cell Res. Ther..

